# Resemblance-Ranking Peptide Library to Screen for Binders to Antibodies on a Peptidomic Scale

**DOI:** 10.3390/ijms23073515

**Published:** 2022-03-23

**Authors:** Felix Jenne, Sergey Biniaminov, Nathalie Biniaminov, Philipp Marquardt, Clemens von Bojničić-Kninski, Roman Popov, Anja Seckinger, Dirk Hose, Alexander Nesterov-Mueller

**Affiliations:** 1 Institute of Microstructure Technology, Karlsruhe Institute of Technology (KIT), 76344 Eggenstein-Leopoldshafen, Germany; felix.jenne@t-online.de; 2 HS Analysis, Haid-und-Neu-Straße 7, 76131 Karlsruhe, Germany; sergey.biniaminov@hs-analysis.com (S.B.); nathalie.biniaminov@hs-analysis.com (N.B.); philipp.marquardt@hs-analysis.com (P.M.); 3 Axxelera, Karl-Floesser-Str. 14, 76189 Karlsruhe, Germany; clemens.kninski@axxelera.com (C.v.B.-K.); roman.popov@axxelera.com (R.P.); 4 Department of Hematology and Immunology, Myeloma Center Brussels, Vrije Universiteit Brussel (VUB), Laarbeeklaan 103, 1090 Jette, Belgium; as@lamkapbio.com

**Keywords:** rituximab, off targeting, ultra-high-density peptide microarrays

## Abstract

A novel resemblance-ranking peptide library with 160,000 10-meric peptides was designed to search for selective binders to antibodies. The resemblance-ranking principle enabled the selection of sequences that are most similar to the human peptidome. The library was synthesized with ultra-high-density peptide arrays. As proof of principle, screens for selective binders were performed for the therapeutic anti-CD20 antibody rituximab. Several features in the amino acid composition of antibody-binding peptides were identified. The selective affinity of rituximab increased with an increase in the number of hydrophobic amino acids in a peptide, mainly tryptophan and phenylalanine, while a total charge of the peptide remained relatively small. Peptides with a higher affinity exhibited a lower sum helix propensity. For the 30 strongest peptide binders, a substitutional analysis was performed to determine dissociation constants and the invariant amino acids for binding to rituximab. The strongest selective peptides had a dissociation constant in the hundreds of the nano-molar range. The substitutional analysis revealed a specific hydrophobic epitope for rituximab. To show that conformational binders can, in principle, be detected in array format, cyclic peptide substitutions that are similar to the target of rituximab were investigated. Since the specific binders selected via the resemblance-ranking peptide library were based on the hydrophobic interactions that are widespread in the world of biomolecules, the library can be used to screen for potential linear epitopes that may provide information about the cross-reactivity of antibodies.

## 1. Introduction

Screening for specific antibody binders at the peptidomic scale is of practical value. They can provide important clues about pathogens in the case of circulating antibodies in the patient’s blood or about potential off-targets for therapeutic antibodies.

There are different approaches to determine antibody binders using a screening approach. For example, tissue cross-reactivity assays based on immunohistochemistry are traditionally performed to identify potential off-target epitopes at the tissue level [1]. Data from such studies do not provide comprehensive information about off-target epitopes but are mandatory in preclinical safety assessments [2,3]. Novel approaches comprise recombinant protein arrays generated either by pipetting them onto a solid support [4] or by direct translation and transcription from DNA arrays [5,6,7]. In this way, the binding of the antibody to the conformational fragments of the proteins, as well as the linear fragments available in the configuration that the proteins take on the surface of the chip, can be identified. Because proteins can change their configuration, for example, during various post-translational modifications [8] or an increase in temperature [9], new linear fragments from the proteome can be available for interactions. They can be identified using short (<10 amino acids) peptide libraries for screening at a higher resolution.

H.B. Larman et al. constructed a synthetic representation of the complete human proteome for the discovery of unreported autoantibodies, which were engineered as peptides on the surface of T7 phage [10]. However, phage display screenings are notorious for the identification of false-positive hits [11]. These emerge for two important reasons: binding to non-target-related materials used during selection and propagation advantages [12]. Often, hits from phage display screening are validated, for example, using peptide microarrays to find invariant amino acids via substitutional analysis [13,14] or to determine the affinity and the serological number of peptide–antibody interactions in peptide array format [15]. Knowledge of invariant amino acids in epitopes contributes to a more targeted search for antigens in proteomic databanks.

To fully display the human peptidome, tens of millions of short peptides are needed. This is even more potentiated if, in future, not only linear but also non-linear targets are to be displayed. Today, such possibilities are provided by the maskless lithographic technique [16,17]. However, a density of 1 million different peptides per square centimeter means focusing the modulated light from the beamer into 10 × 10 µm pixels, including the distance between spots, which leads to the partial radiation overlap of neighboring pixels and reduces the quality of synthesis. Such a problem does not arise in mask lithographic peptide synthesis since the mask adheres tightly to the synthesis surface and the diffraction exposure of neighboring pixels is excluded [18]. However, lithographic peptide synthesis must use and precisely position several hundred masks (one mask per amino acid multiplied by the number of peptide residues: 20 × 10 masks and positioning steps for a ten-mer peptide library) to gain complete combinatorics for a library of interest, which makes such chips very expensive. The pseudo-randomization of the peptide content was proposed by Legutki et al. to minimize the number of masks required for super high-density peptide synthesis [19]. However, this procedure significantly reduces the similarity of the pseudo-random peptide library with the human peptidome. Today, displaying the entire peptidome on a single chip is impossible. However, a smart library design for the reduction mapping of a peptidome can be applied.

The goal of this work is to generate a novel resemblance-ranking library of 160,000 10-meric peptides with the highest resemblance to the whole peptidome and the study of its features. As proof of principle, we investigated the interaction of the resemblance-ranking library with the therapeutic anti-CD20 antibody rituximab (RTX) [20]. The selective binders to rituximab were validated by measuring the corresponding dissociation constants and substitutional analysis.

## 2. Results

### 2.1. Combinatorial Diversity versus Human Peptidome

**Figure 1:**
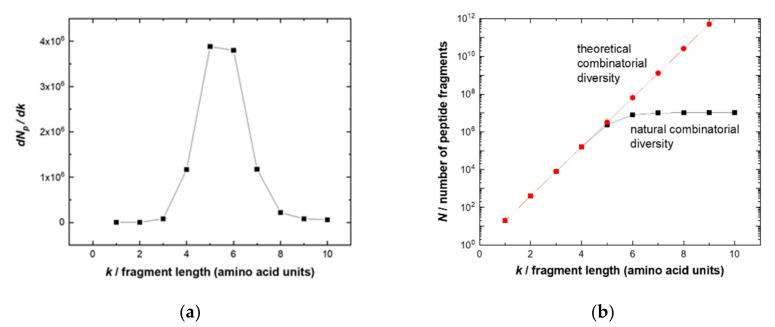
(**a**) Comparison of the number *N* of unique fragments of the complete combinatorial library (red line with circles) with the number *Np* of unique fragments of the human peptidome (black line with squares) depending on the length of the peptide fragment. Starting from the 6-mer peptide, *Np* tends to saturate; (**b**) entropy gain *dNp*/*dk* versus the length k of the peptide sequence.

The theoretical number *N* of unique *k*-mers exponentially grows according to *M^k^*, where *M* = 20 is the number of proteinogenic amino acids. In the human peptidome, however, the diversity of the longer chains with *k* ≥ 7 is significantly reduced and asymptotically approaches 11 million fragments (Figure 1a). This fact determines the optimal length of potential linear epitope fragments, which the adaptive immune system can address.

The likely explanation of such optimization is that, on the one hand, short fragments (*k* ≤ 4) are all present in the peptidome and thus specificity would be difficult to obtain, i.e., resulting in potential autoantigens. On the other hand, targeting longer fragments (*k* > 8) would be more “expensive” (due to the combinatorial complexity of 20*^k^*), necessitating a higher diversity (and therefore number) of naïve B cells. The optimum fragments have a length of five to six amino acids that provide sufficient uniqueness and distinction for a specific sequence between a full-combinatorial library of potential pathogens and a human peptidome. These epitope lengths account for the highest entropy gain of a growing peptide chain (Figure 1b). Thus, the library of 10-mer peptides would be a good approximation to cover the potential linear epitopes of physiologically occurring or therapeutic antibodies relying on the same repertoire. Peptides of this length can also exhibit secondary structures such as alpha helixes (3.4 amino acids per turn) [21].

### 2.2. Design and Properties of the Resemblance-Ranking Peptide Library

In total, 20,350 manually annotated and reviewed proteins of *Homo sapiens sapiens* were collected with the analysis software HSA KIT (HS Analysis GmbH) [22] using the Swiss-Prot section of the UniProt database [23] (access date 27 March 2020). The protein sequences were preprocessed to replace all 37 selenocysteines (U) with cysteines (C). The proteins were computationally sliced into continuous 10-mer fragments with an overlap of nine amino acids. All duplicate fragments were eliminated, resulting in 10,438,489 unique peptides. These peptides, in turn, consisted of 51,475,217 unique k-length sub-fragments (1 ≤ k ≤ 10), which comprised a basis of a high-dimensional vector space.

Each unique peptide was one-hot (binary) encoded [24] as a 51,475,217-dimensional binary vector accounting for all the k-length sub-fragments being included in the respective amino acid sequence. Altogether, the human peptidome was represented as a sparse matrix *SP_nm_* of shape 10,438,489 × 51,475,217, where *n* is the number of unique peptides and *m* the number of unique k-length fragments.

Each encoding k-length sub-fragment was assigned a weighted score according to the number of its occurrences in the whole proteome (see Section 4 and supporting files with codes). These weight scores were represented as vector *W_m_*.

The vector *F_n_* in Equation (1)

(1) $$F_{n}=\overset{m}{\underset{i=1}{\sum }}SP_{ni}W_{i}$$

results as a sum of the weighted scores over all k-length sub-fragments that are included in the corresponding peptide. The peptides with the maximum total weight in the *F_n_* were added to the resemblance-ranking library.

Table 1 presents the ten highest-scored peptides. The difference in scores cannot be used for cross-comparison because they are obtained in different iterations of the sequential scoring and selection algorithm. Each next peptide added to the library has a different basis for evaluation: we reset the scores of those subfragments that are already included in the library with previously selected peptides. Each subsequent peptide is evaluated only for those fragments of *k*-mers that are not yet available in the library.

**Figure 2:**
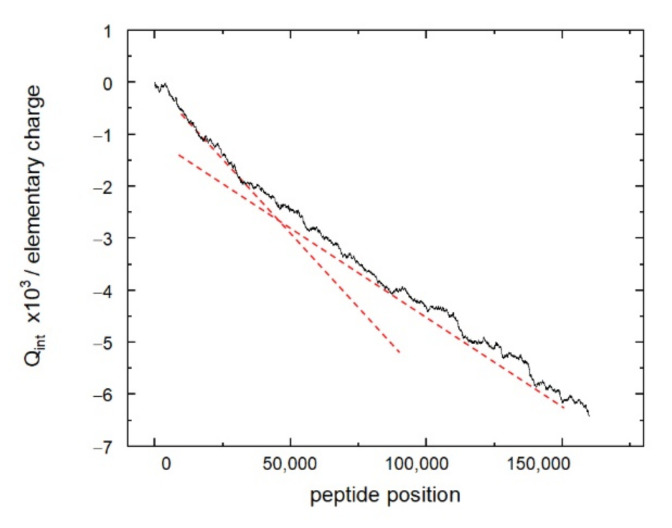
Integral charge of the resemblance-ranking peptide library depending on the interval of integration. Peptides are arranged according to their weight in the library.

The distribution of the peptides of the resemblance-ranking peptide library according to the scores does not correlate with the peptide sum hydrophobicity, sum molecular weight, and sum helix propensity. Interestingly, the resemblance-ranking library has an integral negative charge considering that the occurrence of positively charged amino acids (Arg, His, Lys) is higher than those negatively charged (Asp, Glu) in the peptidome (Figure 2). According to the negative charge, the entire resemblance-ranking library is divided into two groups: the first 30,000 peptides have greater negativity (greater slope of the red dotted line in Figure 2) than the rest.

### 2.3. Interaction of the Resemblance-Ranking Peptide Library with RTX

In the first screen, an RTX concentration of 30 µg/mL was used. This value correlates with a presumptive “active” level of up 20 µg/mL in anti-lymphoma treatment [25,26,27]. We assumed that the strength of the interaction is correlated with the intensity of fluorescent signals from secondary antibodies.

**Figure 3:**
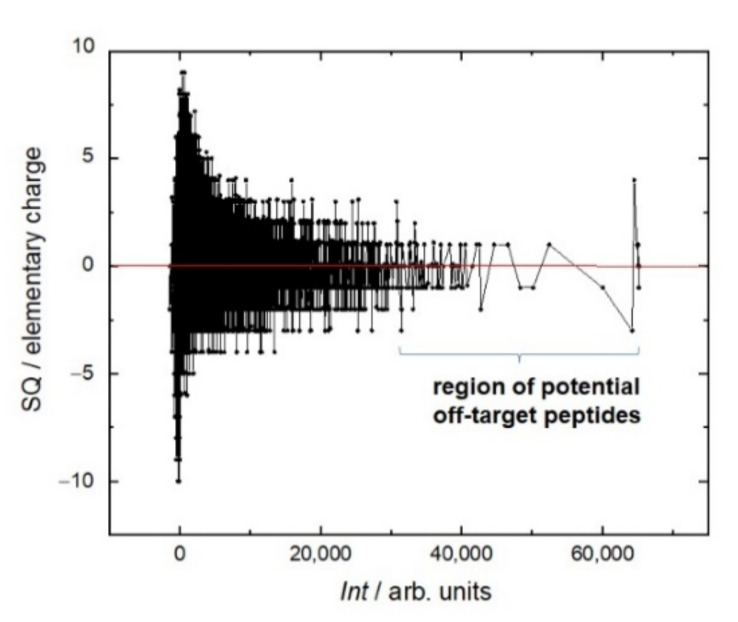
Fluorescent intensity *Int* of RTX interaction with the resemblance-ranking peptide library versus the sum charge SQ of the corresponding peptides. Here and in further graphs, the black line indicates the neighboring peptides with the growing signal intensity.

**Figure 4:**
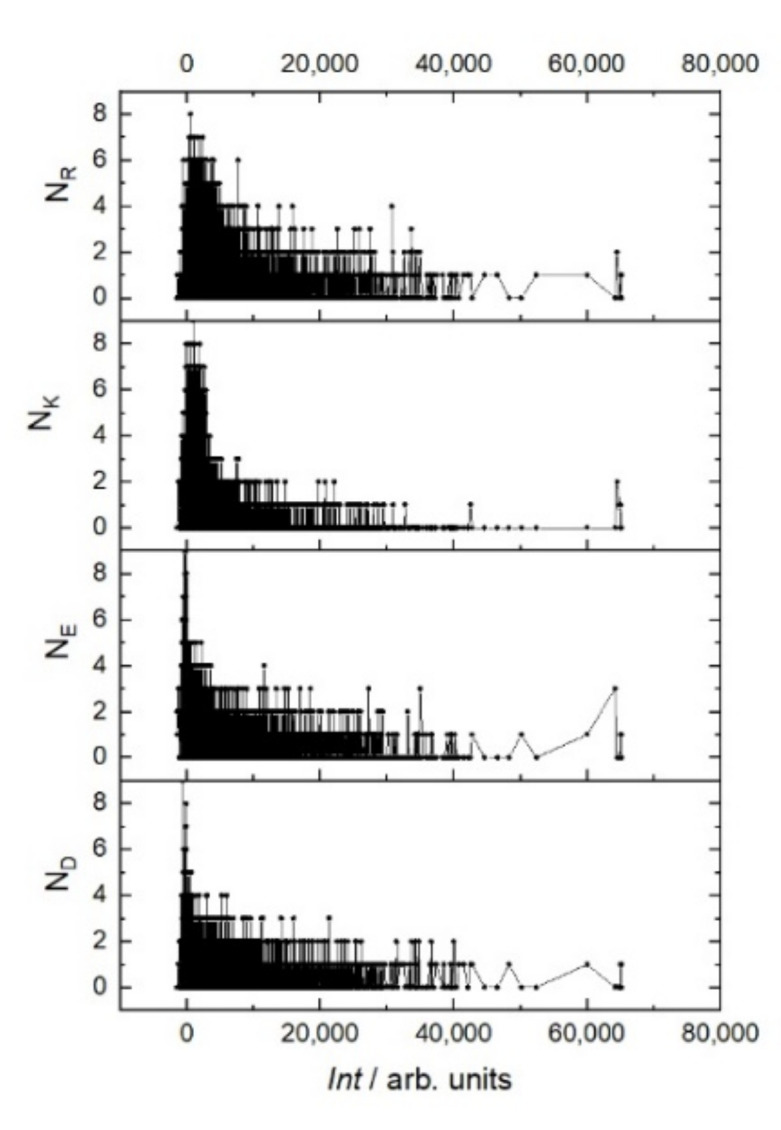
Fluorescent intensity *Int* of RTX interaction with the resemblance-ranking peptide library versus the number N of positively charged amino acids R and K and negatively charged amino acids E and D of the corresponding peptides.

Even though the library itself is integrally negatively charged, during the screen the most intense interaction of RTX was observed with peptides, the total charge of which is no more than two (Figure 3). Moreover, the sign of this charge does not matter. This trend can be seen in the example of the positively charged amino acids arginine (R) and lysine (K) and negatively charged amino acids glutamate (E) and aspartate (D) (Figure 4). In the region of the strongest signals, where off-target sequences are expected, strong charge oscillations are observed, which may reflect the specificity of the rituximab paratope.

**Figure 5:**
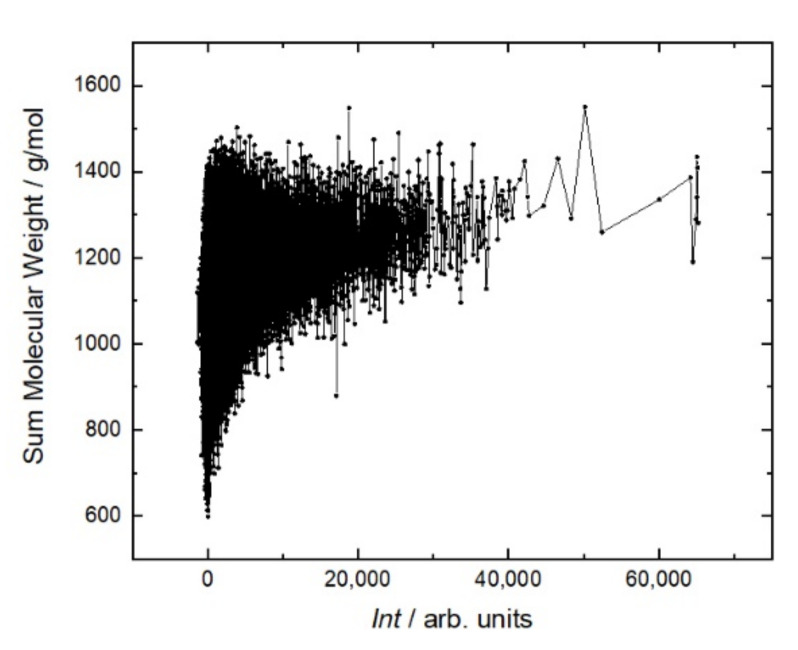
Fluorescent intensity *Int* of RTX interaction with the resemblance-ranking peptide library versus the sum molecular weight of the corresponding peptides.

**Figure 6:**
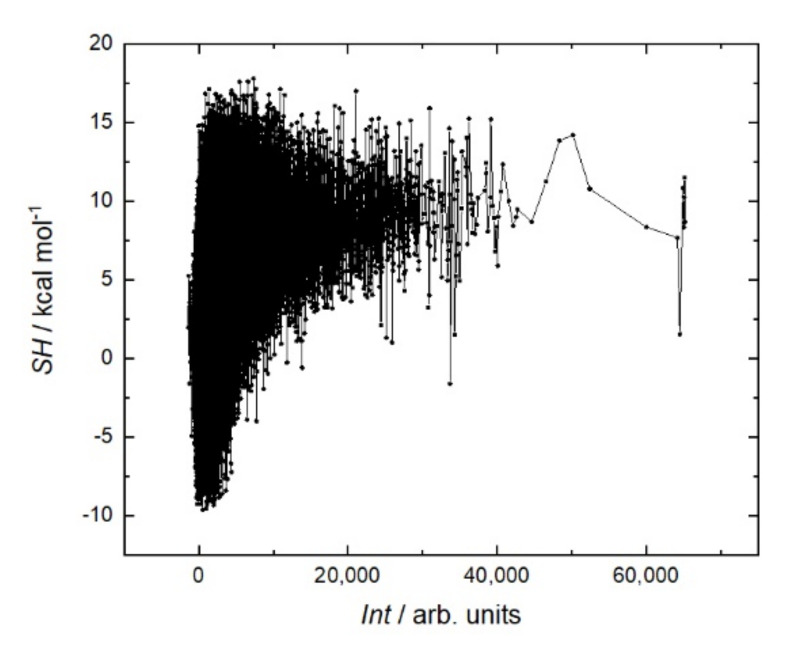
Fluorescent intensity *Int* of RTX interaction with the resemblance-ranking peptide library versus the sum hydrophobicity *SH* of the corresponding peptides [28]. Wimley–White whole-residue hydrophobicity scales were used for calculations.

**Figure 7:**
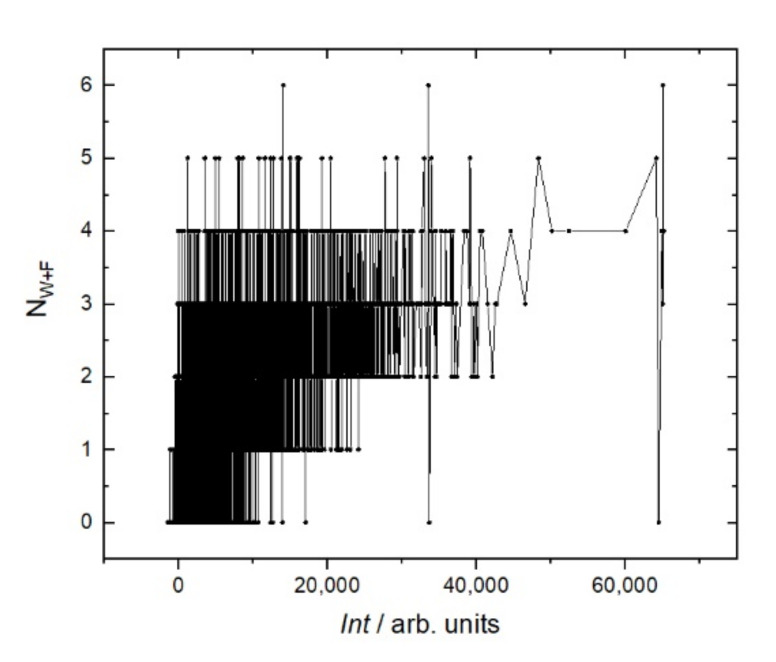
Fluorescent intensity *Int* of RTX interaction with the resemblance-ranking peptide library versus the number of sum of hydrophobic amino acids W and F of the corresponding peptides.

Figure 5 shows that the antibody affinity increases with the molecular weight of peptides. This is due to the appearance of large amino acids, the structures of which are more capable of providing stronger interactions. This is the main mechanism to increase the affinity in the case of linear sequences in contrast to conformational epitopes. As shown in Figure 6, this trend is caused mostly by hydrophobic amino acids. A more detailed analysis of individual amino acids showed that the affinity increases with the number of hydrophobic amino acids, such as tryptophan (W), phenylalanine (F), leucine (L), isoleucine (I), and the polar amino acid tyrosine (Y). These amino acids eliminate the amino acids with low molecular weights, such as alanine (A), threonine (T), and serine (S). Other amino acids, such as methionine (M), valine (V), proline (P), glycine (G), as well as glutamine (Q), and asparagine (N), correlate very weakly with affinity. The increase in the affinity correlates most strongly with the sum of W and F in the peptide (Figure 7).

**Figure 8:**
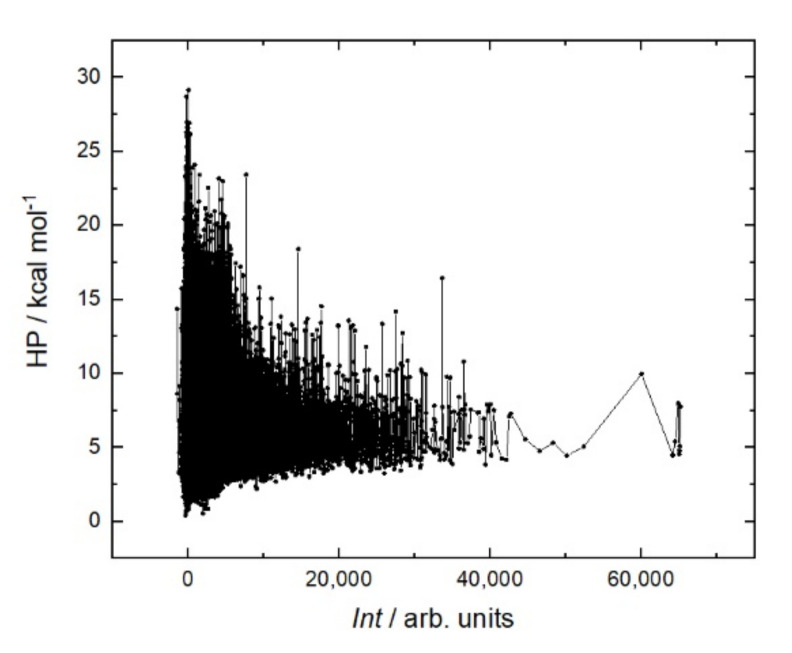
Fluorescent intensity *Int* of RTX interaction with the resemblance-ranking peptide library versus the sum helix propensity *HP* of the corresponding peptides [31].

It is generally believed that a stiffer structure allows for stronger binding in peptide–protein interactions. Various methods are used to reduce entropy; for example, peptide cyclization [29] or the clips method [30]. In the case of rituximab, a sum helix propensity was not a criterion for strong binding to the peptide (Figure 8). On the contrary, the peptides with the strongest affinity are located in the region with a low sum helix propensity of about 5 kcal mol.

### 2.4. Validation of the Resemblance-Ranking Library Hits via Substitutional Microarrays and K_D_ Measurements in Array Format

**Figure 9:**
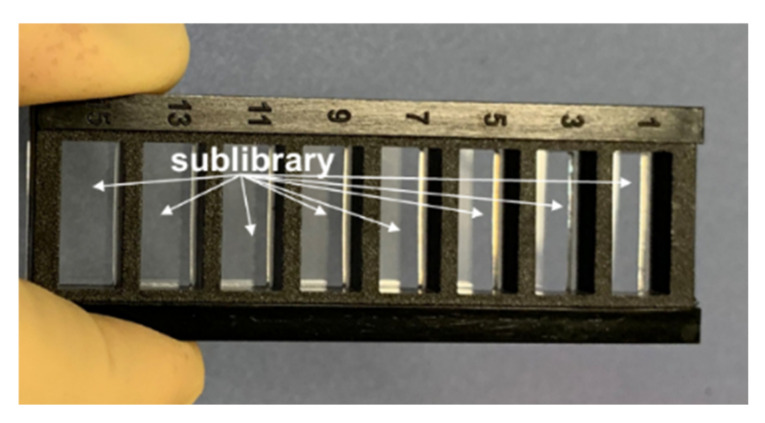
Sublibrary consisting of the substitutions to the peptides selected from the first screens. Eight windows were used to incubate the same sublibrary with different RTX concentrations.

The 30 peptides that gave the highest signals in the first screen were selected to identify dissociation constants *K_D_* to RTX and invariant amino acids. Thanks to the ultra-dense format of peptide microarrays, the library with all substitutions for 30 peptides fit into one of eight windows (Figure 9). Accordingly, eight identical sublibraries were synthesized and incubated with RTX in the form of a diluted series with concentrations of 200, 100, 50, 25, 12.5, 6.25, 3.125, and 1.5625 µg/mL. This allowed the generation of both a substitutional analysis for each selected peptide and to identify individual *K_D_* values for the selected peptides and all 200 substitutional variations for each of the selected peptides. *K_D_* and the maximum fluorescent signal at saturation *Isat* were calculated via the approximation of the measured fluorescent signals by the Formula (2):

(2) $$I_{obs}=I_{sat}\frac{n}{K_{D}+n}$$

where *I_obs_* is the measured signal intensity for the defined spot, *n* is the concentration of the analyte (RTX in our case) in the solution, and *K_D_* is the equilibrium dissociation constant [32].

**Figure 10:**
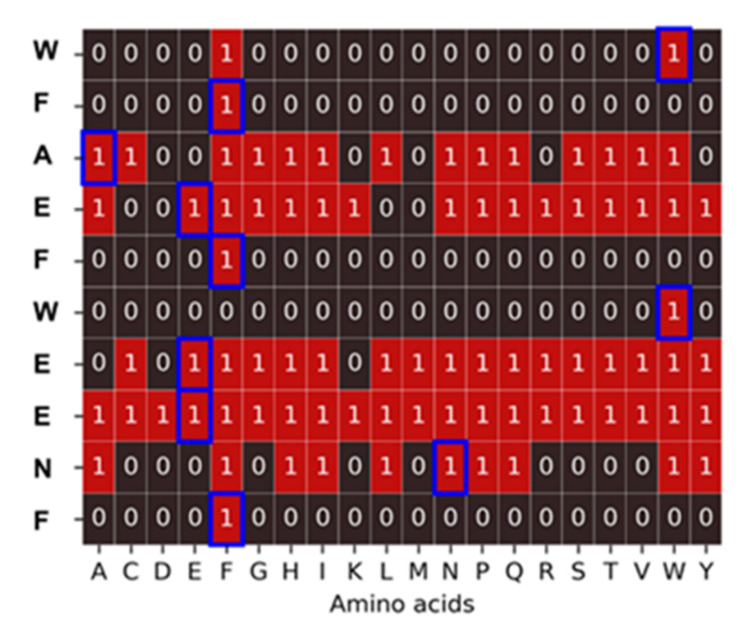
Substitution analysis for the peptide WFAEFWEENF. The blue frames correspond to the amino acids of the peptide under study. Squares show the corresponding fluorescent signal for all substitution sequences. In the case of red squares, the signal is equal to or higher than the signal from the original sequence. The epitope is composed of a combination of the invariant amino acids, W and F: W/FFxxFWxxxF.

In our case, only six peptides had *K_D_* values ≤ 1000 nM (Table 2). These values are significantly higher than the reported *K_D_* for the RTX target, which are between 5 and 20 nM [33]. However, these values are in the range of protein–protein interactions representing important biological functions [34,35]. The selected peptides with *K_D_* ≤ 1000 nM have a significant content of hydrophobic amino acids W and F, which corresponds to the trend shown in Figure 8. Usually, hydrophobic amino acids are associated with nonspecific interactions. Nevertheless, the substitutional analysis showed that epitopes with hydrophobic amino acids have a clear selective profile and may be responsible for binding with high specificity (Figure 10).

## 3. Discussion

The recent analysis of the maturation of the affinity of the capsid protein VP1 of the poliovirus epitope under alanine substitutions showed that there is no clear boundary between the nonspecific and medically relevant sequences [36]. When replacing polyalanine with the corresponding amino acids, the affinity increased in a logarithmic manner up to a known specific signal. The resemblance-ranking library provides a unique opportunity to trace the trends in the formation of selective peptide binders for antibodies, from very weak ones to epitopes of potential significance with *K_D_* values in the submicromolar range.

**Figure 11:**
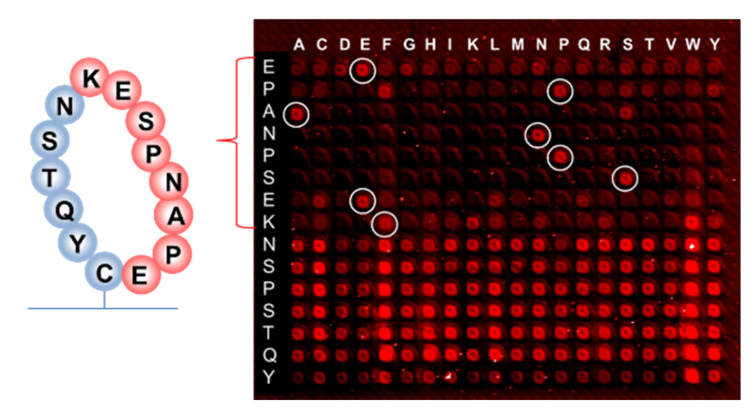
(**right**) Fluorescence scan of the substitutional array for the cyclized peptide EPANPSEKNSPSTQY after incubation with rituximab and subsequent immunostaining. The white circles show amino acids from the original sequence EPANPSEK. The cyclic peptides were formed via the thioether macrocyclization. (**left**) The RTX target sequence and the scheme of the peptide cycle.

Earlier research with phage display suggested that the binding of RTX to the targeted discontinuous epitope EPANPSEKNSSTQY occurs within the extracellular fragment of CD20, cyclized via the disulfide bridge between Cys(167) and Cys(183) residues, which do not contain hydrophobic epitopes [37]. The sequence EPANPSEK was identified as the key region of this interaction using X-ray diffraction [38]. The 10-mer peptide EPANPSEKNS that was presented on the same peptide chip along with the resemblance library gave the signal at the noise level when incubated with RTX (126,756 peptides from the resemblance-ranking library delivered stronger signals). This confirmed the non-linear structure of the RTX target binding. We performed a substitution analysis of the EPANPSEKNSSTQY sequence, which was enclosed in a macrocycle, as shown in Figure 11, to mimic the cyclic fragment of CD20. In this case, the macrocycle was formed using head-to-tail thioether macrocyclization with the orthogonal side group deprotection of cysteine [39].

Although hydrophobic amino acids W and F are not present in the RTX target sequence, selective binding remains when the proline P at the second position of the EPANPSEK epitope is replaced by phenylalanine F, and lysine K at the end of EPANPSEK is replaced by tryptophan W. Other substitutions at these positions resulted in the disruption of the selective binding with RTX.

RTX hydrophobic linear epitopes WWEWS/T [40] and WPXWLE [41] were previously reported. Interestingly, both forward and reverse sequences, WPKWLE and ELWKPW, bind rituximab, but not other anti-CD20 mAbs [42]. Fornoni et al. provided data to support a nonimmune mechanism by which RTX may exert its therapeutic effects in recurrent FSGS after renal transplantation [43]. These data were based on the off-target binding of RTX to the reverse sequence ELWKPW that is part of the acid sphingomyelinase-like phosphodiesterase 3b protein expressed on podocytes. The peptide LWKPWLQPCC from the resemblance-ranking peptide library sharing the 5-meric fragment with this podocyte epitope was identified at the 3156th place in terms of fluorescent signal intensity with a ratio of 0.13 to the maximum signal value among 160,000 peptides. It is likely that medically relevant signals can also be located with the weaker resemblance-ranking library signals within 10% of the maximum fluorescent intensity.

To the best of our knowledge, the molecular reason for the specific epitopes with hydrophobic amino acids is not well understood. At the same time, it is known that water competes with the ligand for binding to the target and critically affects its dissociation [44]. Due to water, binding affinity is often difficult to predict even with structural information [45,46]. It is likely that the observed hydrophobic epitopes carry information about the specific displacement of water molecules from the RTX paratope during ligand association [47].

The resemblance-ranking library does not take into account the concentration of proteins that may be available for interaction with the therapeutic antibody. For the specific target- or off-target binding of monoclonal antibodies, it would indeed be excellent to have data on tissue distribution and an abundance of all potential targets a priori at hand. However, these data do not exist. Even for frequently targeted and best characterized hematological proteins, such as B-cell maturation antigen (BCMA), only semiquantitative data regarding expression height exist [48]. As it is therefore not possible to include target abundances for all potential target sequences across all tissues a priori, we suggest using our approach to first identify potential linear target sequences, and then further assess these regarding target expression and distribution (patho-)physiologically.

## 4. Materials and Methods

### 4.1. Design of the Resemblance-Ranking Peptide Library

To maximize the representing power of the library with 160,000 peptides, only such amino acid sequences were chosen which had the most in common with the whole human peptidome, while being largely different from each other. In each iteration, all 10,438,489 peptides were dynamically scored accounting for the weighted scores of their k-length sub-fragments, as well as for the sub-fragments of the peptides selected in the previous iterations. The initial protein sequences, the resemblance-ranking algorithm as the python codes with comments for every processing step, and the resulting 160,000 peptides are available in the Zenodo repository, https://zenodo.org/record/6046581#.YgaDBJYo82w (access date 20 March 2022).

### 4.2. Staining of the Resemblance-Ranking Peptide Library with RTX

Peptide microarrays: A peptide library of 160,000, peptides as well other peptide microarrays for the substitutions and the *K_D_* analysis, were fabricated via the ultra-high-density peptide microarray technology of AXXELERA (Karlsruhe, Germany) [49]. The AXXELERA technology allows for the 30 µm-sized square peptide spots with a pitch of 60 µm that corresponds to ca. 28,000 spots/cm^2^.

Immunostaining: Peptide microarrays were incubated with the monoclonal anti-CD20 antibody rituximab (Sigma-Aldrich, Steinheim, Germany) with concentrations depending on the type of experiment, diluted in PBS-T (Phosphate Buffered Saline with Tween) overnight at 4 °C. A rituximab concentration of 30 µg/mL was used for the first screen of the entire resemblance-ranking peptide library. Then, the peptide chips were stained with the fluorescently labeled secondary anti-human-IgG antibodies (Jackson ImmunoResearch, Philadelphia, PA, USA). The signals from the spots were read out with the confocal scanner Innoscan 1100 AL. The scans were performed in the red channel (635 nm), with a resolution of 2 µm/pixel, a speed of 35 µm/s, and a PMT gain of 9.

## 5. Conclusions and Outlook

The entropy analysis of the peptidome showed that the five and six amino acid fragments should play an essential functional biological role (constructive, metabolic, and signaling) since they account for the maximum entropy increase. A resemblance-ranking peptide library with 160,000 10-meric peptides that represent the entire human peptidome was designed. The library is based on the vector representation of peptides through their shorter fragments. This approach enables the encoding of the entire peptidome as one large sparse matrix, the dimensions of which are determined by the number of peptides and the number of smaller amino acid fragments. The resemblance-ranking peptide library was constructed from those peptides whose fragments are most frequently present in the peptidome.

The resemblance-ranking library was synthesized using ultra-high-density peptide arrays to search for selective binders to the therapeutic antibody rituximab. The peptide library enabled us to highlight trends in the composition of peptides that enhance their affinity to rituximab. The affinity weakly depended on the charge of the peptide, but it grew with an increase in its mass. This was due to an increase in the number of hydrophobic amino acids, especially tryptophan and phenylalanine. The peptides with the maximum affinity were found in the region with low sum helix propensity.

Dissociation constants *K_D_* were measured for the selected 30 peptides with the highest fluorescent signals. Six peptides with *K_D_* ≤ 1000 nM were identified among them. A substitutional analysis revealed specific epitopes with invariant hydrophobic amino acids W and F.

To show that conformational binders can, in principle, be detected in array format, cyclic peptide substitutions that are similar to the target of rituximab were investigated. Specific substitutions of proline for phenylalanine and lysine for tryptophan in the target rituximab cyclic peptide did not interfere with the selective binding of the antibody. The reason for the selective hydrophobic epitopes was discussed.

The 10-meric peptide binders to rituximab selected in the screens are part of the proteins belonging to the human proteome. This does not mean that the corresponding proteins are automatically off-targeted by the therapeutic antibody. However, information on specific peptide binders can supplement information on off-targets obtained, for example, using other high-throughput methods such as protein arrays, to determine linear epitopes and possible reasons for the cross-reactivity of antibodies.

## Figures and Tables

**Table 1:** Ten highly-scored peptides.

Peptide Sequence	Scores
IHTGEKPYKC	4726
KCEECGKAFS	2006
HQRIHTGERP	1493
THTGEKPYEC	1404
EDEEEEEEED	1299
LPPPPPPPLP	1123
PAAAAAAAGG	1059
EKPYKCEECG	959
PYECKECGKA	956
KPYKCNECGK	906

**Table 2:** Off-target peptides for RTX with a dissociation constant ≤ 1000 nM with their containing proteins.

Off-Target Peptide	*K_D_*, nM	Protein, Submitted Names	ID
WFAEFWEENF	243	Single-stranded DNA-binding protein 3	Q9BWW4
		Metabotropic glutamate receptor 6	O15303
		Metabotropic glutamate receptor 8	O00222
		Seven transmembrane helix receptor	Q8NHA9
		cDNA FLJ75348, highly similar to Homo sapiens metabotropic glutamate receptor 8b	A8K2D2
RDGDRFWWEN	577	Myeloperoxidase	P05164
		Lactoperoxidase	P22079
LHSWWCVFWD	655	Single-stranded DNA-binding protein 2	P81877
		Single-stranded DNA-binding protein 3	Q9BWW4
		Single-stranded DNA-binding protein 4	Q9BWG4
		HSPC116	Q9P038
		Single-stranded DNA binding protein 4, isoform CRA_d	A0A024R7K9
		LisH domain-containing protein	A1L192
SYSLEIQWWY	676	V-set and transmembrane domain-containing protein 2-like protein	Q96N03
		V-set and transmembrane domain-containing protein 2B	A6NLU5
FTGWFLAWDP	853	Villin-1	P09327
		Advillin	O75366
YFPRARWYDY	1110	Probable maltase-glucoamylase 2	Q2M2H8
		Maltase-glucoamylase	Q8TE24
		Maltase-glucoamylase, intestinal	E7ER45

## Data Availability

The initial protein sequences, the resemblance-ranking algorithm as the python codes with comments for every processing step, and the resulted 160,000 peptides are available in the Zenodo repository, https://zenodo.org/record/6046581#.YgaDBJYo82w (access date 20 March 2022).
